# How do people respond to self-test results? A cross-sectional survey

**DOI:** 10.1186/1471-2296-11-77

**Published:** 2010-10-13

**Authors:** Martine HP Ickenroth, Gaby Ronda, Janaica EJ Grispen, Geert-Jan Dinant, Nanne K de Vries, Trudy van der Weijden

**Affiliations:** 1 CAPHRI, School for Public Health and Primary Care, Department of General Practice, Faculty of Health, Medicine and Life Sciences, Maastricht University, P.O.Box 616, 6200 MD Maastricht, The Netherlands; 2 CAPHRI, School for Public Health and Primary Care, Department of Health Promotion, Faculty of Health, Medicine and Life Sciences, Maastricht University, P.O.Box 616, 6200 MD Maastricht, The Netherlands

## Abstract

**Background:**

Self-tests, tests on medical conditions that can be performed by consumers without consulting a doctor first, are frequently used. Nevertheless, there are concerns about the safety of self-testing, as it may delay diagnosis and appropriate treatment in the case of inappropriate use of the test, or false-negative results. It is unclear whether self-tests stimulate appropriate follow-up behaviour. Our aim was to examine the frequency of self-test use, consumers' response to self-test results in terms of their confidence in the result, reassurance by the test result, and follow-up behaviour.

**Methods:**

A two step cross-sectional survey was designed. A random sample of 6700 Internet users in an existing Internet panel received an online questionnaire on the use of self-tests. Self-tests were defined as tests on body materials, initiated by consumers with the aim to diagnose a disease or risk factor. A second questionnaire on consumers' response to self-test results was sent to the respondents that were identified as a self-tester in the first questionnaire (n = 703).

**Results:**

18.1% (799/4416) of the respondents had ever performed a self-test, the most frequently used tests being those for diabetes (5.3%), kidney disease (4.9%), cholesterol (4.5%), urinary tract infection (1.9%) and HIV/AIDS and Chlamydia (both 1.6%). A total of 78.1% of the testers with a normal test result and 81.4% of those with an abnormal result reported confidence in this result. Almost all (95.6%) of the testers with a normal result felt reassured. After a normal result, 78.1% did not take any further action and 5.8% consulted a doctor. The corresponding figures after an abnormal test result were 9.3% and 72.2%, respectively.

**Conclusions:**

Respondents who had performed a self-test seemed to base their follow-up behaviour on the result of the test. They had confidence in the test result, and were often reassured by a normal result. After an abnormal result, most self-testers sought medical care. Because consumers seem to trust the self-test results, further research should focus on the development of consumer information addressing indications for performing a self-test, the validity of self-tests and appropriate interpretation of and management after a test.

## Background

Self-testing, which implies that consumers can decide to test themselves for medical conditions without consulting a doctor first, seems to fit in perfectly with our current views of people taking responsibility for their own health. In recent years, self-testing has become a phenomenon that cannot be ignored. A survey among Dutch Internet users in 2006 showed that 16 percent of all respondents had ever used a self-test[1]. In-vitro self-tests are available for about 25 conditions, for example to detect high cholesterol, HIV or prostate cancer[2]. Self-tests can be bought through the Internet or at a chemist's (home-tests), or are offered and performed by organisations in public places like supermarkets (street-corner tests). Other forms of self-tests are direct-access tests or home collect tests, in which consumers can go to a laboratory (or send body materials to a laboratory) and later receive the results by post or the Internet. Self-tests have regularly attracted media attention in recent years.

We defined a self-test as an in-vitro test on body materials, initiated by a consumer (without consulting a doctor or other health professional), and with the aim of diagnosing a particular disease or identifying a risk factor. We excluded monitoring tests, as they are generally initiated on the advice of a doctor, and pregnancy tests, because these do not detect disease.

Self-testing can offer people a convenient alternative to tests initiated by a doctor. Consumers may perceive fewer barriers to testing, such as embarrassment in the case of HIV or Chlamydia testing[3-5]. It also gives consumers an opportunity to take responsibility for their own health, and could make them conscious of the importance of a healthy lifestyle. Nevertheless, there are concerns about the safety of self-testing, as it may delay diagnosis and appropriate treatment in the case of inappropriate use of the test, or false-negative results[6,7]. For example, a false negative result on a HIV self-test may delay treatment or result in infection of other individuals. It is unclear whether self-tests stimulate appropriate follow-up behaviour. Self-testing might result in a higher burden on the health care system, for example when false-positive results lead to further and more expensive investigations. Hardly any specific research findings on self-testing are currently available. The consequences of self-testing are still unknown, and are the subject of debate[8-12].

Investigating the consequences of self-testing requires research into the extent of the use of self-tests and the perceptions of self-testers with regard to the interpretation of the test result, confidence in the result, reassurance and follow-up behaviour. Our aim was to validate earlier findings about the frequency of self-testing and to investigate consumers' follow-up behaviour after performing a self-test by assessing the actions taken by self-testers. Secondary goals were to describe the confidence self-testers have in self-tests and reassurance experienced after a normal test result.

## Methods

### General design

We designed a cross-sectional two-step questionnaire survey among an existing Internet panel.

### Study population and procedure

Questionnaires on self-testing were sent to an open-access panel managed by Flycatcher, an ISO-certified institute for online research associated with Maastricht University. All persons aged 12 years and older and who have an e-mail address can join the panel. Members are recruited online, by written invitation, face-to-face contacts or by intermediaries. The panellists are invited by e-mail to participate in surveys and they receive a gift voucher when they have completed a certain number of questionnaires. Currently, the Flycatcher Panel consists of people between 12 and 96 years of age, with a mean age of 37 http://www.flycatcher.eu. Compared with the Dutch population, the panellists in the Flycatcher Panel are younger, have a higher level of education and are more often female. The total panel is representative of the Dutch Internet population.

In September 2008, a questionnaire on the use of self-tests was sent to a random sample of 6700 people in the panel. This number was based on the results of an earlier questionnaire on self-testing in 2006[1]. A second questionnaire about follow-up behaviour was sent in November 2008, and was only addressed to those respondents who were identified by the first questionnaire as being self-testers. On further consideration, we excluded self-testers who had performed an ovulation test, because this test is often used by healthy persons, rather than by those who want to detect infertility disorders. We asked self-testers questions about one specific self-test they had mentioned in the first questionnaire. If respondents had mentioned multiple tests, a hierarchical selection procedure was applied to select one of the tests they had used, in order to collect information on different types of self-tests. Both questionnaires were online for 2 weeks; non-responders were sent a reminder after one week.

### Ethical approval

The Medical Ethical Committee of Maastricht University indicated that no ethical approval was needed for this study.

### Variables

The first questionnaire that was sent to the Flycatcher panel contained questions on whether people had ever heard of self-tests, and whether they had ever performed a self-test or were likely to perform a self-test in the future (Additional file 1). We also asked for socio-demographic characteristics. The questionnaire had been developed by Ronda et al. and has already been used to describe the use of self-tests in 2006[1].

The second questionnaire was newly developed for consumers who had ever used a self-test, and contained questions on confidence in the test result, reassurance and follow-up behaviour (Additional file 2). This questionnaire was based on the results of earlier research[13], and on consensus among the research team. We asked self-testers questions on the following topics:

- Respondent characteristics. Did the respondents have certain complaints at the time they performed the self-test (yes, no)?

- Test result. Was the result of the test normal (negative test result), abnormal (positive test result) or inconclusive?

- Confidence in test result and reassurance. Were they reassured by a normal test result (options: yes, completely reassured; yes, partly reassured or no, not reassured) and did they have confidence in the test result? (I have confidence in the result of the self-test, answered on a 5-point scale: totally agree, agree, neutral, disagree and totally disagree).

- Follow-up behaviour after the self-test. The next topic was about management related to the test result. Respondents could select multiple options: no further action, consulted a doctor, consulted another health care professional (e.g. dietician, physician assistant, doctor's assistant), changed lifestyle, used complementary medicine (e.g. acupuncture), bought self-medication, searched for more information, discussed result with family or friends, performed a self-test for other diseases or risk factors, or other. In the case of a positive test result, we added an extra question for those respondents who had not taken any action after the test result, and asked them why (options: already been diagnosed with the disease, did not trust test result or not knowing what to change or what to do).

If a respondent had consulted a doctor, we asked what kind of doctor (options: general practitioner, specialist at hospital, doctor at a municipal health service or other). We also asked them to indicate their most important reason for consulting a doctor, from the following options: to discuss complaints, to discuss concerns about having a disease, to ask for more information about the test or the disease, to discuss the test result, to ask for a new test, to ask for other tests or for referral to a hospital. In the case of a positive test result, another option was to receive treatment.

Final questions were whether they had told their doctor that they had performed the test, and whether they had received treatment or had been referred to a hospital.

### Statistical analysis

Basic descriptive statistics were used to describe the respondents' characteristics, the use of self-tests, and consumers' response to self-test results in terms of their confidence in the result, reassurance by the test result, and follow-up behaviour. Answers to the question whether respondents were reassured by the test result were recoded into yes (totally reassured and partly reassured) or no (not reassured). Confidence in the test result was recoded into having confidence (I agree or totally agree with having confidence in the test result), neutral, or not having confidence in the test result (disagree or totally disagree). Chi-square and Fisher exact tests were used to assess differences in follow-up behaviour between self-testers with a normal test result and self-testers with an abnormal test result. Differences were considered to be statistically significant if p < 0.05 (two-sided). Analyses were performed with SPSS (Version 16.0).

## Results

**Figure 1:**
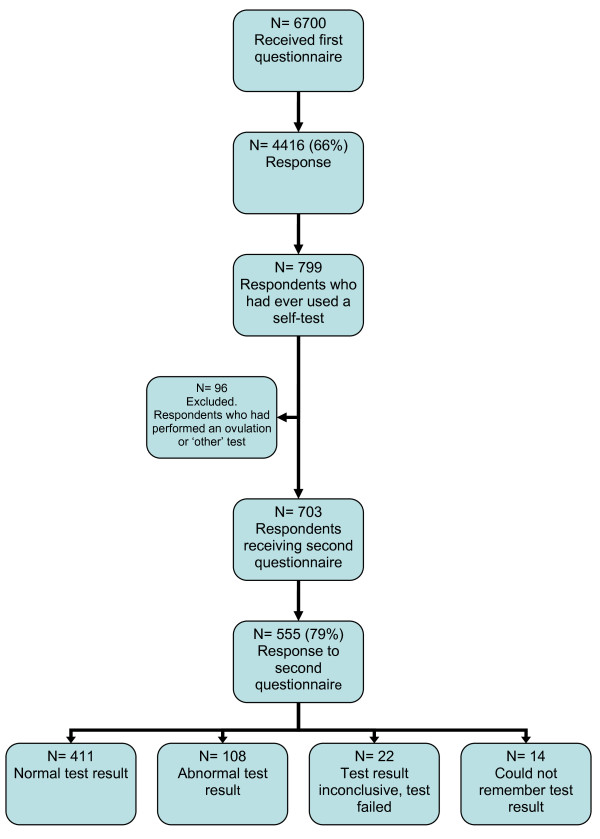
**Respondents to the questionnaires sent in September 2008 (first questionnaire) and November 2008 (second questionnaire)**.

The response to the first survey in September 2008 was 66% (n = 4416) (Figure 1). Of the respondents in 2008, 2613 had completed a similar questionnaire in 2006 as well; the other 1803 respondents had received the questionnaire for the first time in 2008. The November 2008 questionnaire with questions on follow-up behaviour was sent to 703 self-testers, 555 of whom responded (response rate 79%). Self-testers who had performed an ovulation test or respondents who had performed a test marked as 'other', were not addressed in the second questionnaire (n = 96).

### Respondents' characteristics and frequency of self-test use

The mean age of the respondents in September 2008 (n = 4416) was 40.2 years (SD 14.1). Sixty-four percent of the respondents were female. Twenty-one percent had a low level of education (primary school, lower general secondary education or lower vocational education), 42% an intermediate level (higher general secondary education or intermediate vocational education), and 36% a high level of education (higher vocational education or university).

The group of self-testers (n = 555) had a mean age of 42.2 years (SD 14.0), and 70.5% of the self-testers were female. Seventeen percent of the self-testers had a low level of education, 43.4% an intermediate level, and 39.8% a high level of education.

The percentage of respondents who had ever performed at least one self-test was 18.1% (n = 799), and 73% of the self-testers had performed their most recent self-test in the past two years. The most commonly used self-tests were those to detect diabetes (n = 232, 5.3% of all respondents), kidney disease (n = 216, 4.9%), cholesterol (n = 198, 4.5%), urinary tract infection (n = 85, 1.9%), Chlamydia (n = 71, 1.6%) and HIV/AIDS (n = 71, 1.6%) (Table 1). Forty-four percent of all self-tests had been performed as true home tests. The self-tests to detect kidney disease, ovulation tests and tests for female fertility were most often used as true home tests (Table 1).

**Table 1:** Number and percentage of respondents who had ever performed a self-test (multiple entries possible) and frequency of true home testing

	N	% of all respondents (N = 4416)	% true home tests*
**Total**	**799**	**18.1**	**44**

Diabetes	232	5.3	56
Kidney disease	216	4.9	92
Cholesterol	198	4.5	23
Urinary tract infection	85	1.9	46
AIDS/HIV	71	1.6	9
Chlamydia	71	1.6	3
Ovulation**	53	1.2	98
Allergies	49	1.1	14
Anaemia	48	1.1	13
Syphilis	38	0.9	8
Hepatitis B/C	37	0.8	3
Female fertility	35	0.8	80
Glandular fever	34	0.8	29
Vaginal infection/candida	34	0.8	12
HPV	18	0.4	17
Influenza	17	0.4	65
Pharyngitis	16	0.4	50
Male fertility	14	0.3	29
Thyroid disease	12	0.3	8
Blood coagulation	9	0.2	33
Osteoporosis	8	0.2	13
Hereditary disease	6	0.1	0
Prostate cancer	5	0.1	40
Intestinal cancer	3	0.1	67
Gluten intolerance	2	0.04	50
Helicobacter pylori	2	0.04	50
Liver disease	2	0.04	0
Other**	93	2.1	60

* Percentage of the tests that were performed as true home tests

* * Tests excluded in second questionnaire

### Test result, reassurance and confidence in test result

Overall, 411 respondents had received a negative test result (normal test result), 108 had tested positive (abnormal test result) and 22 reported that the test had failed or that the test result had been inconclusive (Figure 1).

Of the self-testers with a normal test result, 78.1% (n = 321) reported they had confidence in this result, while 3.4% (n = 14) had no confidence in the result. Almost 96% (n = 393) of the testers were reassured by the normal result.

In the case of an abnormal test result, 81.4% (n = 88) of the respondents had confidence in this result. Almost 4% (n = 4) of the self-testers who tested positive did not have confidence in the result.

### Follow-up behaviour based on the test result

#### Normal test result

Self-testers who had a normal test result (n = 411) generally had not taken any further action (n = 321, 78.1%) (Table 2). Six percent of the respondents (n = 24) with a normal test result had consulted a doctor, and 1.0% (n = 4) had consulted another health professional. The other respondents had engaged in self-management, for example by changing their lifestyle (n = 20, 4.9%), or searched for more information (n = 16, 3.9%). Eight percent of the respondents who had a normal test result (n = 33) had discussed the result with friends or family. None of the respondents had decided to perform self-tests for other diseases after the first test.

**Table 2:** Follow-up behaviour based on the test result

Action (%)	No further action	Consulted doctor	Consulted other health professional	Changed lifestyle	Comple- mentary medicine	Self-medication	Searched for more information	Consulted friends/family	Other
All self-tests									
- normal (N = 411)	78.1**	5.8**	1.0**	4.9**	1.0**	0.5**	3.9**	8.0*	3.4
- abnormal (N = 108)	9.3	72.2	8.3	18.5	7.4	10.2	17.6	17.6	2.8

Diabetes									
- normal (N = 86)	76.7	8.1	3.5	5.8	0	1.2	4.7	7.0	2.3
- abnormal (N = 20)	15.0	80.0	20.0	25.0	5.0	5.0	15.0	10.0	0
Kidney disease									
- normal (N = 104)	92.3	1.9	0	0	0	0	0	3.8	1.9
- abnormal (N = 6)	33.3	50.0	0	0	0	0	16.7	33.3	16.7
Cholesterol									
- normal (N = 97)	79.4	7.2	0	5.2	0	0	7.2	9.3	2.1
- abnormal (N = 20)	5.0	80.0	10.0	35.0	5.0	0	10.0	10.0	0
Urinary tract infection									
- normal (N = 22)	63.6	13.6	4.5	13.6	9.1	0	0	9.1	0
- abnormal (N = 35)	5.7	85.7	2.9	2.9	2.9	2.9	2.9	2.9	0
Chlamydia									
- normal (N = 39)	69.2	0	0	7.7	0	0	0	15.4	7.7
- abnormal (N = 3)	33.3	33.3	0	0	0	0	33.3	33.3	33.3
HIV									
- normal (N = 18)	72.2	0	0	11.1	5.6	0	5.6	16.7	5.6
- abnormal (N = 1)	0	100	0	100	0	0	100	100	0
Allergies									
- normal (N = 12)	58.3	8.3	0	8.3	8.3	0	16.7	25.0	0
- abnormal (N = 7)	0	14.3	14.3	57.1	42.9	57.1	57.1	42.9	14.3

* P = 0.003

** P < 0.001

#### Abnormal test result

After an abnormal test result (n = 108), most respondents had sought medical care (72.2% (n = 78) consulting a doctor and 8.3% (n = 9) another health professional). Nine percent of the self-testers with an abnormal test result (n = 10) had not taken any further action despite the abnormal result. Reasons for not taking further action included already having been diagnosed with the disease (n = 5) or not knowing what to do or what to change (n = 2). Others had engaged in self-management. Almost 19% of the self-testers had changed their lifestyle (n = 20), and 18% had told others about the self-test they had performed (n = 19). Other forms of self-management used after performing a self-test were self-medication and complementary medicine. The differences in follow-up behaviour between a normal and an abnormal test result were all statistically significant (Table 2).

Self-test follow-up behaviour seemed to be similar for most tests. After the self-test to detect allergies (n = 19) respondents seemed to be more likely to engage in self-management than after the other tests. They had used self-medication or complementary medicine, or had changed their lifestyle. Only one person had consulted a doctor. The urinary tract infection self-test had been performed by 61 respondents, of which 22 had a normal and 35 an abnormal test result. Almost all of these testers had been having complaints (52/57). All respondents who tested negative had been reassured by this result, and only 13.6% of them had consulted a doctor. A positive test result led to a consultation in 85.7% of all cases.

### Reasons for consulting a doctor and medical treatment

Of the self-testers with a normal test result (n = 411), 24 (5.8%) had decided to consult a doctor, despite the normal test result. Their reasons for consulting a doctor are shown in table 3. Almost all self-testers with a normal test result had told the doctor about the self-test they had performed (n = 22). Of the 24 respondents who had consulted a doctor, 12 had received treatment and 5 had also been referred to a hospital.

**Table 3:** Reasons for consulting a doctor after normal and abnormal self-test result (multiple entries possible)

	Abnormal test result N = 108	Normal test result N = 411
Consulted doctor	N = 78 (72.2%)	N = 24 (5.8%)

To discuss complaints	41	9
For treatment	31	0
To discuss the self-test result	27	8
To ask for a new test	16	6
To discuss concerns about having a disease	6	6
To receive more information about a disease	4	0
To receive more information about the self-test	2	4
To ask for other tests	2	2
To ask for referral to a hospital	2	0

Of the self-testers with an abnormal test result (n = 108), 78 (72.2%) had consulted a doctor (Table 3). Almost all of these respondents had told their doctor they had performed a self-test (n = 73). Of the 78 respondents with an abnormal test result who had consulted a doctor, 59 had received treatment, of which 16 were also referred to a hospital and 5 were only referred to a hospital.

## Discussion

### Main findings

In 2008, 18.1% of the respondents in our Internet survey had ever performed a self-test. The most frequently used self-tests were those to detect diabetes, kidney disease, high cholesterol, urinary tract infections, HIV/AIDS and Chlamydia. Respondents who had performed a self-test seemed to base their follow-up behaviour on the test result. Most of the self-testers had confidence in the test result. In the case of a normal test result, they had generally been reassured by this result, and had not taken any further action. After an abnormal test result, many had sought medical care, engaged in self-medication, searched for more information or changed their lifestyle. Follow-up behaviour seemed to be similar for most self-tests, except for a self-test on allergies, after which the respondents seemed to be more likely to engage in self-management than after other tests. This might be explained by the test specific properties of a self-test to detect allergies; after a positive result, consumers can adjust their lifestyle, a consultation with a doctor is often not necessary.

### Strengths and limitations

Using the Flycatcher Internet panel, we were able to send our questionnaires to a large sample of Dutch Internet users. Because this sample is not completely representative of the Dutch population, it might overestimate the frequency of self-testing. Internet users may be more interested in self-testing, since they will probably more often search the Internet for health related questions, and therefore can be more informed of the existence of self-tests and more willing to use one. Another reason for a possible overestimation of the frequency of self-testing is because women are overrepresented in the panel. Women are more likely to engage in self-testing.[14] Additionally, some self-tests are gender specific tests (e.g. ovulation tests) or are more often used by women (e.g. urinary tract infection self-test). We think the overestimation due to respondents using monitoring tests (e.g. glucose testing in diabetes) instead of diagnostic self-tests is small. Secondary analysis showed that only 22 respondents who had performed a diabetes self-test were actually diabetics.

Our results do not allow us to conclude what the exact consequences of self-testing are at individual level or for health care providers. For example, we do not know whether self-testing leads to overconsultation of doctors because of increased anxiety among consumers with a positive result (which may be false-positive), or whether self-tests effectively help consumers diagnose a disease or identify a risk factor at an earlier stage, and can prevent disease or complications. To determine these consequences, the whole process of self-testing should be investigated, starting with the way each consumer decided to do a test, and ending with verifying the diagnosis with a gold standard clinical investigation and the actual follow-up behaviour of the self-tester. The steps in the self-testing process involve challenging decisions for consumers to make, for example deciding whether there is a medical indication for the test, dealing with possible false-positive or false-negative results, and seeing the results in the light of multiple risk factors, for example in cardiovascular disease. Although we cannot say whether all self-testers made the right decisions during this process, we can conclude that consumers seem to rely on self-test results, and that they should be informed about the pitfalls and possible consequences of self-testing.

### Comparison with other studies

We compared our data on the prevalence of self-testing with the results of the survey done in 2006. The use of self-tests seems to be stable, as the only slight increase was seen in the use of the Chlamydia self-test and a test to detect kidney disease. Both of these tests have been offered for free to the Dutch population in the past two years, accompanied by a mass media campaign. For example, a self-test to detect renal disease which was provided for free by the Dutch kidney association, was ordered by 7.8% of the Dutch population during the first thirty days[15].

Respondents in our study seemed to have confidence in the test result. Even when they had symptoms (e.g. in the urinary tract infection self-test) they often relied on the test result. Since we found no published articles on consumers' confidence in self-tests, we cannot compare our findings with those of other studies. Qualitative research in general practice showed that patients have high expectations of medical lab tests[16], and consumers may think likewise about self-tests.

## Conclusions

Our research assessed the actual actions taken by consumers who had performed a self-test. Further details on consumers' belief in self-test results, the way they interpret self-test results and whether and how they engage in certain follow-up behaviours should be investigated in qualitative research. This could also shed more light on the psychological and medical consequences of self-testing.

It is almost impossible to answer the question whether self-tests should be either encouraged or prohibited, the pros and cons of self-testing will always have to be weighed for each test and each individual. Consumers should be able to weigh the pros and cons themselves and make an informed decision. As consumers have a high level of confidence in self-tests, and are reassured by a negative test result, it is very important that consumers have adequate information on the reliability and the validity of self-tests in general, that tests can give false positive and false negative results, and how consumers should interpret test results. Health professionals should be able to provide this information, but this should also be available on the Internet, since self-testing often does not involve a health professional.

Further research should further focus on information that explains to consumers for whom it is important to do a test, as well as information that consumers need to correctly perform a self-test, so they can make informed choices when they intend to do a self-test, and have sufficient knowledge to respond suitably to the test result.

## Competing interests

The authors declare that they have no competing interests.

## Authors' contributions

MI, GR, JG and TW were involved in all aspects of the study. GD and NV had a consultative role during the study. MI analysed the data and drafted the paper. All other authors contributed and commented on drafts and approved the final version.

## Pre-publication history

The pre-publication history for this paper can be accessed here:

http://www.biomedcentral.com/1471-2296/11/77/prepub

## Supplementary Material

Additional file 1 **Questionnaire 1**. Translation of the questionnaire that was sent in September 2008 Click here for file

Additional file 2 **Questionnaire 2**. Translation of the questionnaire that was sent to self-testers in November 2008. This questionnaire also contains questions on information use and needs. In this article, we only focused on the questions on follow-up behaviour. Click here for file
