# Supplementary material for: How do people respond to self-test results? A cross-sectional survey
Source: BMC Fam Pract. 2010 Oct 13;11:77. doi: 10.1186/1471-2296-11-77 (PMC2964597; doi:10.1186/1471-2296-11-77)
Supplement: Additional file 1 — Questionnaire 1. Translation of the questionnaire that was sent in September 2008 [file 1471-2296-11-77-S1.DOC]

**Self-tests**

**September 2008**

**The questionnaire was originally in Dutch and has been translated into English**

More and more tests are becoming available that allow you to examine your own health. In other words, tests that don’t have to be ordered by a doctor. Not much is known about the current use of such tests in the Netherlands and the need for them. In our survey we try to investigate the use of self-tests en find out what factors may influence their use.

What do we mean by self-tests in this survey?

#### By self-test we mean a test on body samples (such as blood, urine, faeces or saliva) that can be used to detect a disease or the risk of getting a disease, and which you carry out, or have carried out, at your own initiative (so not on the advice of your own doctor).

What do we NOT consider a self-test in this survey?

- A blood pressure meter is not a self-test in this sense, as it uses no body materials.
- Pregnancy tests are excluded from this survey since pregnancy is not a disease.
- A glucose test which you use at home to monitor your blood sugar levels because you have Diabetes, are NOT considered a self-test, because this is not aimed at detecting a disease. The disease is already known.
- To ask your doctor to perform a test is NOT considered a self-test because you have a consult with your doctor before doing the test. We do ask questions about this later on.

There are several ways to use self-tests:

1. buying a self-test for home use from a chemist, pharmacy or supermarket, or ordering it by post, via the Internet or a newspaper or magazine coupon, then applying the test yourself en reading the results;

2. visiting some facility (which may also be a supermarket) at your own initiative, having a test done there and getting the results immediately;

3. visiting a laboratory (e.g. at a hospital) at your own initiative, having a test done there, and getting results sent to you by post;

4. sending in a body sample to a laboratory (at your own initiative), where they do a test and send you the results by post.

It is important that the decision to have the test was taken by you, and not by your doctor.

#### **Questions about self-tests that can be used to detect a disease or the risk of a disease**

**1. Before this survey, had you ever heard of self-tests? (if yes, multiple answers allowed)**

- yes, I’d heard of self-tests for home use
- yes, I’d heard about visiting a facility, having a test done there, and getting the results immediately
- yes, I’d heard about visiting a laboratory to have a body sample taken, and getting the results sent to me by post
- yes, I’d heard about sending in a body sample to a laboratory, and getting the test results sent to me by post
- no (respondent is referred to question 4a)

**2. Have you ever considered using a self-test? (if yes, multiple answers allowed)**

- yes, a self-test for home use
- yes, visiting a facility, having a test done there and getting the results immediately
- yes, visiting a laboratory to have a body sample taken, and getting the results sent to me by post
- yes, sending in a body sample to a laboratory, and getting the test results sent to me by post
- no (respondent is referred to question 4a)

**3a1. Have you ever done a self-test / had a self-test done? (if yes, multiple answers allowed)**

- No
- Yes, namely…..
- Diabetes
- Cholesterol
- Allergies (hay fever, asthma, house dust mite, food allergies)
- Urinary tract infection
- Aids / HIV
- Anaemia (haemoglobin, hb)
- Ovulation
- Chlamydia
- Glandular fever
- Hepatitis B or C
- Female fertility or menopause
- Male fertility
- Syphilis
- Vaginal infection (Candida, vaginitis)
- Kidney diseases
- Thyroid diseases
- Influenza
- Blood coagulation
- Intestinal cancer
- Prostate cancer (PSA)
- Cervical cancer (HPV)
- Helicobacter pylori
- Gluten intolerance (celiac disease)
- Pharyngitis
- Liver diseases
- Osteoporosis
- Hereditary diseases
- Other tests, namely…….

3a2. Were these tests part of a larger set of tests?

|  | Yes | No |
| --- | --- | --- |
| Cholesterol | ○ | ○ |
| Diabetes | ○ | ○ |
| etc. [only the answers to question 3a are shown in the table] | ○ | ○ |

**3b. When was the last time** you did this/these self-test(s)?

|  | Less than 2 years ago | More than 2 years ago |
| --- | --- | --- |
| Cholesterol | ○ | ○ |
| Diabetes | ○ | ○ |
| etc. [only the answers to question 3a are shown in the table] | ○ | ○ |

***3c. What was the result of this self-test?***

|  | Normal  (nothing wrong) | Abnormal (something wrong) | Inconclusive | Test failed | Can’t remember | Would rather not say |
| --- | --- | --- | --- | --- | --- | --- |
| Cholesterol | ○ | ○ | ○ | ○ | ○ | ○ |
| Diabetes | ○ | ○ | ○ | ○ | ○ | ○ |
| etc. [only the answers to question 3a are shown in the table] | ○ | ○ | ○ | ○ | ○ | ○ |

**3d**. Where did you buy / have this/these self-test(s)?

|  | Test for home use | Had it done at supermarket, chemist’s, pharmacy or sports centre (e.g. in a screening van) | Visited laboratory to have sample taken | Sent in body sample to a laboratory | Other |
| --- | --- | --- | --- | --- | --- |
| cholesterol | ○ | ○ | ○ | ○ | ○ |
| diabetes | ○ | ○ | ○ | ○ | ○ |
| etc. [only answers from question 3a listed in table] | ○ | ○ | ○ | ○ | ○ |

**3d1. Where did you buy or get** the self-test?

|  | Chemist’s | Pharmacy | Supermarket | Internet | Newspaper or magazine | Other |
| --- | --- | --- | --- | --- | --- | --- |
| Cholesterol | ○ | ○ | ○ | ○ | ○ | ○ |
| Diabetes | ○ | ○ | ○ | ○ | ○ | ○ |
| etc. [only the answers to question 3a are shown in the table] | ○ | ○ | ○ | ○ | ○ | ○ |

3e. Was the test free or did you pay for it?

|  | Free | Paid for it myself | Reimbursed by insurance | Other |
| --- | --- | --- | --- | --- |
| Cholesterol | ○ | ○ | ○ | ○ |
| Diabetes | ○ | ○ | ○ | ○ |
| etc. [only the answers to question 3a are shown in the table] | ○ | ○ | ○ | ○ |

3f. Was this test part of a (national or local) campaign?

|  | No | Yes | Don’t know |
| --- | --- | --- | --- |
| Cholesterol | ○ |  | ○ |
| Diabetes | ○ |  | ○ |
| etc. [only the answers to question 3a are shown in the table] | ○ |  | ○ |

**3g1. You have indicated that the cholesterol self-test was part of a (national or local) campaign. Can you indicate below which organization was responsible for this campaign?**

………………………………

**4a. Do you intend to use self-tests in the future?**

- Definitely not (respondent is referred to question 5)
- Probably not (respondent is referred to question 5)
- Perhaps
- Probably
- Definitely

**4b. What self-test(s) would you consider? (multiple answers allowed)**

- Diabetes
- Cholesterol
- Allergies (hay fever, asthma, house dust mite, food allergies)
- Urinary tract infection
- Aids / HIV
- Anaemia (haemoglobin, hb)
- Ovulation
- Chlamydia
- Glandular fever
- Hepatitis B or C
- Female fertility or menopause
- Male fertility
- Syphilis
- Vaginal infection (Candida, vaginitis)
- Kidney diseases
- Thyroid diseases
- Influenza
- Blood coagulation
- Intestinal cancer
- Prostate cancer (PSA)
- Cervical cancer (HPV)
- Helicobacter pylori
- Gluten intolerance (celiac disease)
- Pharyngitis
- Liver diseases
- Osteoporosis
- Hereditary diseases
- Other tests, namely…….

**4c. What type(s) of self-test would you consider? (multiple answers allowed)**

- A self-test for home use
- Visiting a facility, having a test done there, and getting the results immediately
- Visiting a laboratory to have a body sample taken, and getting the results sent to me by post
- Sending in a body sample to a laboratory, and getting the results sent to me by post

**5a. In the past two years, did you ever ask your family doctor to have a particular test done on your materials (like blood)? (If yes, multiple answers allowed)**

- No
- Yes, namely….
- Diabetes
- Cholesterol
- Allergies (hay fever, asthma, house dust mite, food allergies)
- Urinary tract infection
- Aids / HIV
- Anaemia (haemoglobin, hb)
- Ovulation
- Chlamydia
- Glandular fever
- Hepatitis B or C
- Female fertility or menopause
- Male fertility
- Syphilis
- Vaginal infection (Candida, vaginitis)
- Kidney diseases
- Thyroid diseases
- Influenza
- Blood coagulation
- Intestinal cancer
- Prostate cancer (PSA)
- Cervical cancer (HPV)
- Helicobacter pylori
- Gluten intolerance (celiac disease)
- Pharyngitis
- Liver diseases
- Osteoporosis
- Hereditary diseases
- Other tests, namely…….

**5b Was this test actually done at the time?**

|  | Yes | No |
| --- | --- | --- |
| Cholesterol | ○ | ○ |
| Diabetes | ○ | ○ |
| etc. [only the answers to question 5a are shown in the table] | ○ | ○ |

**6. Do you intend to ask your family doctor to do a test (or do the same test again) in the future?**

- Certainly not
- Probably not
- Possibly
- Probably
- Certainly

Here are some more questions about your health status, lifestyle and personal details.

#### **Health status**

#### **7. How would you rate your own health?**

- Very poor
- Poor
- Reasonable
- Good
- Very good

**8. Do you have any chronic disease, impairment or disability?**

- Yes: …
- No

#### **Lifestyle factors**

**9. Have you smoked one or more cigarettes (including roll-your-owns), cigars of pipes in the last 7 days?**

- Yes
- No

**10. On how many days a week are you physically active (e.g. walking, cycling, sports, gardening, or physically demanding household chores like mopping, vacuuming and cleaning windows) for at least 30 minutes a day?**

… days a week [respondents can enter a maximum of 1 digit between 0 and 7]

**11. On how many days a week do you eat at least 200 g (4 serving spoons) of vegetables?**

… days a week [respondents can enter a maximum of 1 digit between 0 and 7]

**12. On how many days a week do you eat at least 2 pieces of fruit?**

… days a week [respondents can enter a maximum of 1 digit between 0 and 7]

**13. On how many days a week do you take the type and quantities of fat in various products into account when deciding what to eat?**

… days a week [respondents can enter a maximum of 1 digit between 0 and 7]

**14. On how many days a week do you usually drink beer, wine or other alcoholic beverages?**

… days a week [respondents can enter a maximum of 1 digit between 0 and 7]

**15. How many glasses do you usually drink on such a day? If you don’t drink any alcohol, please fill in 0.**

… glasses a day [respondents can enter only figures]

**16. Do you use any nutritional supplements (extra vitamins and/or minerals and suchlike)?**

- Yes
- No

**17. Do you use any homeopathic medicines to increase your disease resistance or improve your health?**

- Yes
- No

**18. Are you, or have you ever been, a blood donor?**

- Yes, I’m currently a blood donor
- Yes, I’ve donated blood in the past
- No, I’ve never donated blood

# Demographic factors

**19. What is your height (in centimetres)?**

… cm [respondents can enter a maximum of 3 digits between 100 and 250]

**20. What is your weight (in kilogrammes)?**

… kg [respondents can enter a maximum of 3 digits between 0 and 999]

**21. What nationality do you have?**

- Dutch
- Belgian
- German
- Moroccan
- Surinam
- Turkish
- Other: …

**22.** **What is your home situation?**

- I live with my parent(s) / carer(s) / guardian(s).
- I live alone.
- I live together with my partner, without children.
- I live together with my partner and child(ren).
- I live with my child(ren) without a partner.
- I live in a home for the elderly / nursing home.
- I share a house with other people (e.g. fellow-students, brother or sister, etc.).
