# Supplementary material for: How do people respond to self-test results? A cross-sectional survey
Source: BMC Fam Pract. 2010 Oct 13;11:77. doi: 10.1186/1471-2296-11-77 (PMC2964597; doi:10.1186/1471-2296-11-77)
Supplement: Additional file 2 — Questionnaire 2. Translation of the questionnaire that was sent to self-testers in November 2008. This questionnaire also contains questions on information use and needs. In this article, we only focused on the questions on follow-up behaviour. [file 1471-2296-11-77-S2.DOC]

**Self-tests**

**Version:** B – done self-test, Dept. of General Practice

**Date:** 22 October 2008_definitive

Some time ago, you indicated on a questionnaire that you had used a …[dynamic representation] self-test (*indicate form*). The questions below are about this self-test and various aspects associated with it.

The first questions ask you for general information on the self-test you did. This is followed by a number of questions about the various phases of the self-testing process:

1. before you take the test;
2. the actual use of the test (only for tests done at home);
3. interpreting the test result;
4. what to do with the test result.

The heading at the top of each page indicates what phase of the process the questions are about.

#### **Questions specifically about the** [dynamic representation] **self-test**

**1a. In what form did you take the [dynamic respresentation] self-test?**

- I did the self-test myself, at home.
- I went to a particular place (supermarket, pharmacy, chemist’s), had the test done there and was given the results immediately.
- I went to a laboratory, where they took some body materials, and I then received the results at home by mail, e-mail or through the internet.
- I sent off body materials to a laboratory and received the results at home by mail, e-mail or through the internet.

**1b. When did you take this self-test?**

- - - 0-3 months ago
    - 4- 6 months ago
    - 7-12 months ago
    - Over 12 months ago

**1c. What was the main reason for you to do a [dynamic representation] self-test?**

- - - Because I had a medical complaint.
    - Because I was worried that I might have a disease.
    - Because other people advised me to take the test.
    - Because people in my immediate environment had the disease.
    - Because I had had the test before and wanted to know if anything had changed.
    - Because I wanted to know more about my health status.
    - Other, namely ………………………………..

**1d. Why did you decide to take a self-test?** *(multiple answers allowed)*

- - - Because such a test ensures privacy.
    - Because you get the results quickly.
    - Because the test was offered to me (free of charge).
    - Because I wanted to take responsibility for my own health.
    - Because I previously asked my doctor to do the test but it was never done.
    - Other, namely…………………………………………………..

**1e. What was the result of the self-test?**

- Normal (nothing wrong)
- Abnormal (something wrong)
- Inconclusive
- Test failed
- Can’t remember

**The following questions are about the information you heard or read before you decided to do the self-test (or have it done)**

**2a. Before you tested yourself (or had yourself tested), had you heard or read any information about this test?**

- Yes, I had heard some information (proceed to 2c).
- Yes, I had read some information (proceed to 2b).
- Yes, I had heard and read some information (proceed to 2b).
- No (proceed to 2h).

**2b. What part of the information did you read?**

- I read all of the information.
- I looked at the headings and only read the parts that were interesting and/or relevant to me.
- I only read the beginning and/or the end of the information.
- I read some sentences here and there in the text to decide if the rest of it was interesting and/or relevant to me.

**2c. How did you get the information?**

- I asked for it myself or looked it up (proceed to 2d)
- Through others (proceed to 2e)
- I came upon it by accident (proceed to 2e)
- Other, namely ……….. (proceed to 2e)

**2d. Were you able to find the information you were looking for?**

- Yes
- No

**2e. Where or from whom did you get this information? (multiple answers allowed)**

- From relatives, friends or colleagues
- From my family doctor, from a specialist
- At my family doctor’s office (e.g. from a brochure)
- At the pharmacy
- At the chemist’s
- From TV or radio
- From newspapers, magazines or books
- From the Internet
- At the supermarket
- Other, namely…..

**2f. What was the information about? (multiple answers allowed)**

- For whom the test is intended
- How the test should be carried out
- What disease or risk factor the test identifies
- The reliability of the test result
- Where the test is done or sold
- The meaning of the test result
- The costs of the test, and if they are reimbursed by my insurance
- Whether I can have the test done at my family doctor’s
- Other, namely…….

**2g. What was your general opinion about the information you heard or read**?

| 1 Very poor | 2 | 3 | 4 | 5 very good |
| --- | --- | --- | --- | --- |
| 1 very hard to understand | 2 | 3 | 4 | 5 very easy to understand |
| 1 Very unclear | 2 | 3 | 4 | 5 very clear |
| 1 very unrelaible | 2 | 3 | 4 | 5 very reliable |
| 1 Very incomplete | 2 | 3 | 4 | 5 very complete |
| 1 Very difficult | 2 | 3 | 4 | 5 very easy |

**2h. In what way would you prefer to get information about self-tests? (multiple answers allowed)**

- From relatives, friends or colleagues
- From my family doctor, from a specialist
- At my family doctor’s office (e.g. from a brochure)
- At the pharmacy
- At the chemist’s
- From TV or radio
- From newspapers, magazines or books
- From the Internet
- At the supermarket
- Other, namely…...

**2i. What would be important for you to know before you do the test? (multiple answers allowed)**

- For whom the test is intended
- How the test should be carried out
- What disease or risk factor the test identifies
- The reliability of the test result
- Where the test is done or sold
- The meaning of the test result
- The costs of the test, and if they are reimbursed by my insurance
- Whether I can have the test done at my family doctor’s
- Other, namely…….

**The following questions are only intended for people who have done tests at home**

**The questions below are about the information you have used or read about the way you actually have to perform the test.**

**Some questions refer to the patient information leaflet; by this we mean the instructions for use included in the packaging of the test.**

**3a. Did you read the information on the packaging before you bought the test?**

- - Yes, all of it
  - Yes, part of it
  - No

**3b. Did you read (or re-read) the information on the packaging before you did the test?**

- - Yes, all of it
  - Yes, part of it
  - No

**If the answer to 3a AND 3b was ‘No’, proceed to question 3f; otherwise 3c**

**3c. Do you think the information on the packaging helped you to better perform the test?**

- Yes
- No

**3d. Open question: could you indicate what information you thought was missing from the packaging?...................................................................................................**

**3e. What was your general opinion of the information on the packaging**?

| 1 Very poor | 2 | 3 | 4 | 5 Very good |
| --- | --- | --- | --- | --- |
| 1 Very hard to understand | 2 | 3 | 4 | 5 Very easy to understand |
| 1 Very unclear | 2 | 3 | 4 | 5 Very clear |
| 1 Very unreliable | 2 | 3 | 4 | 5 Very reliable |
| 1 Very incomplete | 2 | 3 | 4 | 5 Very complete |
| 1 Very difficult | 2 | 3 | 4 | 5 Very easy |

**3f. Did you read the information in the patient information leaflet about the way to perform this self-test?**

- Yes, all of it
- Yes, part of it
- No (proceed to 3j)

**3g. In your opinion, did the information in the patient information leaflet help you to better perform the test?**

- Yes
- No

**3h. Open question: Could you indicate what information about performing the test was missing from the patient information leaflet?.......................................................................**

**3i. What was your general opinion of the information on performing the test that was given in the patient information leaflet**?

| 1 Very poor | 2 | 3 | 4 | 5 Very good |
| --- | --- | --- | --- | --- |
| 1 Very hard to understand | 2 | 3 | 4 | 5 Very easy to understand |
| 1 Very unclear | 2 | 3 | 4 | 5 Very clear |
| 1 Very unreliable | 2 | 3 | 4 | 5 Very reliable |
| 1 Very incomplete | 2 | 3 | 4 | 5 Very complete |
| 1 Very difficult | 2 | 3 | 4 | 5 Very easy |

**3j. Did you look for or get any other information about the way to perform the test besides the information on the packaging and/or in the patient information leaflet?**

- Yes, I looked for it (proceed to 3k)
- Yes, I got it (proceed to 3l)
- No (proceed to the questions about performing the test)

**3k. Did you actually find the information you were looking for?**

- Yes
- No

**3l. Where or from whom did you seek this additional information and where or from whom did you get it?**

- From relatives, friends or colleagues
- From my family doctor, from a specialist
- At my family doctor’s office (e.g. from a brochure)
- At the pharmacy
- At the chemist’s
- From TV or radio
- From newspapers, magazines or books
- From the Internet
- At the supermarket
- Other, namely…...

**3m. Do you think this additional information helped you to better perform the test?**

- Yes
- No, everything was already in the patient information leaflet
- No, it did not help me
- Other, namely……………………………….

**3n. Open question: Could you indicate what information about performing the test was missing from the additional information you got?...............................................................**

**The following questions are on the information you used or read about how to interpret the test result, in other words, about the meaning of the test result.**

**4a1. For tests used at home: were you able to see the test result clearly?**

- Yes
- No

**4a2. For tests used at home: did you read the information about the meaning of your test result in the patient information leaflet?**

- Yes (proceed to 4c)
- No (proceed to 4f)

**4a3. For lab tests and street-corner tests: were you given information about the meaning of your test result when you received the result?**

- Yes (proceed to 4b)
- No (proceed to 4f)

**4b. Did you read this information?**

- Yes, all of it
- Yes, part of it
- The information was given to me orally
- No

**4c. What was your general opinion on the information about the meaning of your test result**?

| 1 Very poor | 2 | 3 | 4 | 5 Very good |
| --- | --- | --- | --- | --- |
| 1 Very hard to understand | 2 | 3 | 4 | 5 Very easy to understand |
| 1 Very unclear | 2 | 3 | 4 | 5 Very clear |
| 1 Very unreliable | 2 | 3 | 4 | 5 Very reliable |
| 1 Very incomplete | 2 | 3 | 4 | 5 Very complete |
| 1 Very difficult | 2 | 3 | 4 | 5 Very easy |

**4d. Do you think this information helped you to better interpret your test result?**

- Yes
- No

**4e. Open question: Could you indicate what information about interpreting your test result was missing?..................................................................................**

**4f. Did you look for or get any additional information about interpreting your test result?**

- Yes, I looked for it (proceed to 4g)
- Yes, I received it (proceed to 4h)
  - No (please continue with question 5)

**4g. Did you actually find the additional information you were looking for?**

- Yes
- No

**4h. Where or with whom did you look for this additional information and where or from whom did you get it?**

- From relatives, friends or colleagues
- From my family doctor, from a specialist
- At my family doctor’s office (e.g. from a brochure)
- At the pharmacy
- At the chemist’s
- From TV or radio
- From newspapers, magazines or books
- From the Internet
- At the supermarket
- Other, namely…...

.

**4i. Do you think this additional information helped you to better interpret your test result?**

- Yes
- No, everything was already in the patient information leaflet
- No, it did not help me
- Other, namely……………………………….

**4j. Open question: Could you indicate what additional information you would have liked to have to better interpret your test result but couldn’t get?...........................................................**

**4k. What was your general opinion on the additional information about interpreting your test result**?

| 1 Very poor | 2 | 3 | 4 | 5 Very good |
| --- | --- | --- | --- | --- |
| 1 Very hard to understand | 2 | 3 | 4 | 5 Very easy to understand |
| 1 Very unclear | 2 | 3 | 4 | 5 Very clear |
| 1 Very unreliable | 2 | 3 | 4 | 5 Very reliable |
| 1 Very incomplete | 2 | 3 | 4 | 5 Very complete |
| 1 Very difficult | 2 | 3 | 4 | 5 Very easy |

**The following questions are about the results of this self-test.**

**5. To what extent do you agree with the following statements about the self-test?**

| **Statement** | **Completely disagree** | **Disagree** | **Neither agree nor disagree** | **Agree** | **Completely agree** |
| --- | --- | --- | --- | --- | --- |
| I have confidence in the results of this self-test. |  |  |  |  |  |
| If the test result is normal (nothing is wrong) you can assume that this test result is reliable. |  |  |  |  |  |
| If the test result is abnormal (something is wrong), you can assume that this result is reliable. |  |  |  |  |  |
| I have just as much confidence in this self-test as in a test ordered by a doctor. |  |  |  |  |  |
| I would recommend this self-test to others. |  |  |  |  |  |
| This self-test offers me certainty about my own health. |  |  |  |  |  |

**The following questions are about your test result and the follow-up steps you may have taken as a consequence of the result.**

**If the test result was normal (nothing wrong)**

**6. Did you have any medical complaints when you did this self-test?** *(loop: question is skipped if complaints were reason to take self-test (1c))*

- - Yes
  - No

**7. Did you repeat the test to make sure?**

- - - Yes
    - No

**8. Were you reassured by the result of this self-test?**

- - Yes, completely
  - Yes, partly
  - No

**9. What did you do after you got the test result?** *(multiple answers allowed)*

- - - I took no further action.
    - I discussed the test result with relatives or friends.
    - I looked for further information.
    - I did a self-test for other diseases or risk factors.
    - I changed my lifestyle.
    - I bought over-the-counter drugs (such as vitamins or pills).
    - I sought help from complementary medicine (such as acupuncture or homeopathy).
    - I consulted a doctor.
    - I consulted another care provider (such as a dietician, a psychologist, a physiotherapist, my GP’s receptionist or a nurse practitioner).
    - Other

**9a. *If looked for further information:* Where did you look for further information?**

- At my family doctor’s office (e.g. from a brochure)
- At the pharmacy
- At the chemist’s
- From TV or radio
- From newspapers, magazines or books
- From the Internet
- At the supermarket
  - Other

**9b. *If lifestyle changed:* What lifestyle changes did you make? *(multiple answers allowed*)**

- - - Took some rest
    - Avoided stress / took things a bit easier
    - Started to eat healthier food / changed my diet
    - Used less alcohol
    - Gave up smoking
    - Took more exercise
    - Had safe sex
    - Bought over-the-counter drugs
    - Avoided things I’m allergic to
    - Other

**9c. *If doctor was consulted:* What kind of doctor did you consult?**

- - A general practitioner
  - A specialist at the hospital
  - A doctor at the regional public health service
  - Other

*Next questions only if a doctor was consulted*

**10. What was your main reason for consulting a doctor?**

- - I wanted to discuss my medical complaint with the doctor.
  - I wanted to discuss my concern about having a particular disease or health risk.
  - I wanted to ask for further information about the test.
  - I wanted to ask for further information about the disease for which I used the test.
  - I wanted to discuss my test result with a doctor.
  - I wanted the doctor to repeat the test.
  - I wanted the doctor to do other tests.
  - I wanted to be referred to hospital.

**10a *If doctor was asked to repeat the test:* Why did you want the test to be repeated by the doctor?**

- - I did not trust the result of the self-test.
  - I wanted more certainty about the result of the self-test.
  - I was not sure if I had done the test correctly.
  - I was not sure if this self-test was the right one to find out about the disease or the risk.

**11. Did you tell the doctor you had done a self-test?**

- - - Yes
    - No

**11a. *If answer to question 11 was Yes:* Did the doctor explain to you what the result of the self-test meant for your situation?**

- - Yes
  - No

**12. Did the doctor repeat the same test you did as a self-test, or order the same test to be done, for instance at the hospital?**

- - Yes
  - No

**12a. *If answer to question 12 was Yews:* Was the result of your self-test the same as that of the test done or ordered by the doctor?**

- - - Yes
    - No

**13. Did the doctor order any other tests?**

- - Yes
  - No

**14. Did the doctor reassure you?**

- - - Yes, completely
    - Yes, partly
    - No

**15. Were you satisfied with what the doctor did or told you?**

- - Yes, completely satisfied
  - Yes, partly satisfied
  - Neither satisfied nor dissatisfied
  - No partly dissatisfied
  - No, completely dissatisfied

**16. Did the doctor give you any treatment because of the result of the self-test?**

- - - Yes, he gave me treatment for the disorder I had tested myself for
    - Yes, but for a different disorder
    - No

**17. Did the doctor refer you to hospital as because of the results of the self-test?**

- - Yes, for the disorder I had tested myself for
  - Yes, but for a different disorder
  - No

*For all respondents:*

**18. Are you currently being treated for the disorder for which you tested yourself?**

- - Yes
  - No

**If test result was abnormal (something wrong)**

**6. Did you have any medical complaints when you did the self-test?** *(loop: question is skipped if complaints were reason to take self-test)*

- - Yes
  - No

**7. Did you repeat the test to make sure?**

- - - Yes
    - No

**8. What did you do after you got the test result? (Multiple answers allowed)**

- - - I took no further action.
    - I discussed the test result with relatives or friends.
    - I looked for further information.
    - I did a self-test for other diseases or risk factors.
    - I changed my lifestyle.
    - I bought over-the-counter drugs (such as vitamins or pills).
    - I sought help from complementary medicine (such as acupuncture or homeopathy).
    - I consulted a doctor.
    - I consulted another care provider (such as a dietician, a psychologist, a physiotherapist, my GP’s receptionist or a nurse practitioner).
  - Other

**8a. *If no further action was taken:* Why did you take no further action?**

- - - I already knew about the disease or the risk.
    - I did not trust the test result.
    - I did not know what to change / was unable to change anything.

***8b If further information was sought:* Where did you look for further information?**

- At my family doctor’s office (e.g. from a brochure)
- At the pharmacy
- At the chemist’s
- From TV or radio
- From newspapers, magazines or books
- From the Internet
- At the supermarket
  - Other

**8c *If lifestyle was changed:* What lifestyle changes did you make? *(Multiple answers allowed)***

- - - Took some rest
    - Avoided stress / took things a bit easier
    - Started to eat healthier food / changed my diet
    - Used less alcohol
    - Gave up smoking
    - Took more exercise
    - Had safe sex
    - Bought over-the-counter drugs
    - Avoided things I’m allergic to
  - Other

**8d *If doctor was consulted:* What kind of doctor did you consult?**

- - A general practitioner
  - A specialist at the hospital
  - A doctor at the regional public health service
    - Other

*Next questions only if a doctor was consulted*

**9. What was your main reason for consulting a doctor?**

- - I wanted to discuss my medical complaint with the doctor.
  - I wanted to discuss my concern about having a particular disease or health risk.
  - I wanted to ask for further information about the test.
  - I wanted to ask for further information about the disease for which I used the test.
  - I wanted to discuss my test result with a doctor.
  - I wanted the doctor to repeat the test.
  - I wanted the doctor to do other tests.
  - I wanted to get treatment for the disease or health risk I had discovered by means of the test.
  - I wanted to be referred to hospital.

**9a *If doctor was asked to repeat the test:* Why did you want the test to be repeated by the doctor?**

- - I did not trust the result of the self-test.
  - I wanted more certainty about the result of the self-test.
  - I was not sure if I had done the test correctly.
    - I was not sure if this self-test was the right one to find out about the disease or the risk.

**10. Did you tell the doctor you had done a self-test?**

- - Yes
  - No

**10a *If answered to question 10 was Yes:* Did the doctor explain to you what the result of the self-test meant for your situation?**

- - - Yes
    - No

**11. Did the doctor repeat the same test you did as a self-test, or order the same test to be done, for instance at the hospital?**

- - Yes
  - No

***11a. If answer to question 11 was Yes:* Was the result of your self-test the same as that of the test done or ordered by the doctor?**

- - - Yes
    - No

**12. Did the doctor order any other tests?**

- - Yes
  - No

**13. Did the doctor reassure you?**

- - - Yes, completely
    - Yes, partly
    - No

**14. Were you satisfied with what the doctor did or told you?**

- - Yes, completely satisfied
  - Yes, partly satisfied
  - Neither satisfied nor dissatisfied
  - No, partly dissatisfied
  - No, completely dissatisfied

**15. Did the doctor give you any treatment because of the result of the self-test?**

- - - Yes, he gave me treatment for the disorder I had tested myself for
    - Yes, but for a different disorder
    - No

**16. Did the doctor refer you to hospital as because of the results of the self-test?**

- - Yes, for the disorder I had tested myself for
  - Yes, but for a different disorder
  - No

*For all respondents:*

**17. Are you currently being treated for the disorder for which you tested yourself?**

- - Yes
  - No

**If test failed / inconclusive / can’t remember**

**6. Did you have any medical complaints when you did the self-test?** *(loop: question is skipped if complaints were reason to take self-test)*

- - Yes
  - No

**7. Did you repeat the test to make sure?**

- - - Yes
    - No

**8. What did you do after you got the test result? (Multiple answers allowed)**

- - - I took no further action.
    - I discussed the test result with relatives or friends.
    - I looked for further information.
    - I did a self-test for other diseases or risk factors.
    - I changed my lifestyle.
    - I bought over-the-counter drugs (such as vitamins or pills).
    - I sought help from complementary medicine (such as acupuncture or homeopathy).
    - I consulted a doctor.
    - I consulted another care provider (such as a dietician, a psychologist, a physiotherapist, my GP’s receptionist or a nurse practitioner).
  - Other

**8a. *If further information was sought:* Where did you look for further information?**

- At my family doctor’s office (e.g. from a brochure)
- At the pharmacy
- At the chemist’s
- From TV or radio
- From newspapers, magazines or books
- From the Internet
- At the supermarket
  - Other

**8b. *If lifestyle was changed:* What lifestyle changes did you make? *(Multiple answers allowed)***

- - - Took some rest
    - Avoided stress / took things a bit easier
    - Started to eat healthier food / changed my diet
    - Used less alcohol
    - Gave up smoking
    - Took more exercise
    - Had safe sex
    - Bought over-the-counter drugs
    - Avoided things I’m allergic to
  - Other

**8c. *If doctor was consulted:* What kind of doctor did you consult?**

- - A general practitioner
  - A specialist at the hospital
  - A doctor at the regional public health service
    - Other

*Next questions only if a doctor was consulted*

**9. What was your main reason for consulting a doctor?**

- - Because the test had failed or the result was unclear.
  - I wanted to discuss my medical complaints with the doctor.
  - I wanted to discuss my concern about having a particular disease or health risk.
  - I wanted to ask for further information about the test.
  - I wanted to ask for further information about the disease for which I used the test.
  - I wanted to discuss my test result with a doctor.
  - I wanted a doctor to repeat the test.
  - I wanted a doctor to do other tests.
  - I wanted to be referred to hospital.

**10. Did you tell the doctor you had done a self-test?**

- - Yes
  - No

**11. Did the doctor repeat the same test you did as a self-test, or order the same test to be done, for instance at the hospital?**

- - Yes
  - No

**12. Did the doctor order any other tests?**

- - Yes
  - No

**13. Did the doctor reassure you?**

- - - Yes, completely
    - Yes, partly
    - No

**14. Were you satisfied with what the doctor did or told you?**

- - Yes, completely satisfied
  - Yes, partly satisfied
  - Neither satisfied nor dissatisfied
  - No, partly dissatisfied
  - No, completely dissatisfied

**15. Did the doctor give you any treatment because of the result of the self-test?**

- - - Yes, he gave me treatment for the disorder I had tested myself for
    - Yes, but for a different disorder
    - No

**16. Did the doctor refer you to hospital as because of the results of the self-test?**

- - Yes, for the disorder I had tested myself for
  - Yes, but for a different disorder
  - No

*For all respondents:*

**17. Are you currently being treated for the disorder for which you tested yourself?**

- - Yes
  - No
